# Validation study on definition of cause of death in Japanese claims data

**DOI:** 10.1371/journal.pone.0283209

**Published:** 2023-03-23

**Authors:** Fumiya Ito, Shintaro Togashi, Yuri Sato, Kento Masukawa, Kazuki Sato, Masaharu Nakayama, Kenji Fujimori, Mitsunori Miyashita

**Affiliations:** 1 Department of Palliative Nursing, Health Sciences, Tohoku University Graduate School of Medicine, Sendai, Japan; 2 Division of Integrated Health Sciences, Department of Nursing for Advanced Practice, Nagoya University Graduate School of Medicine, Nagoya, Japan; 3 Department of Medical Informatics, Tohoku University Graduate School of Medicine, Sendai, Japan; 4 Center for the Promotion of Clinical Research, Tohoku University Hospital, Sendai, Japan; 5 Department of Healthcare Administration, Tohoku University Graduate School of Medicine, Sendai, Japan; Bach Mai Hospital, VIET NAM

## Abstract

Identifying the cause of death is important for the study of end-of-life patients using claims data in Japan. However, the validity of how cause of death is identified using claims data remains unknown. Therefore, this study aimed to verify the validity of the method used to identify the cause of death based on Japanese claims data. Our study population included patients who died at two institutions between January 1, 2018 and December 31, 2019. Claims data consisted of medical data and Diagnosis Procedure Combination (DPC) data, and five definitions developed from disease classification in each dataset were compared with death certificates. Nine causes of death, including cancer, were included in the study. The definition with the highest positive predictive values (PPVs) and sensitivities in this study was the combination of “main disease” in both medical and DPC data. For cancer, these definitions had PPVs and sensitivities of > 90%. For heart disease, these definitions had PPVs of > 50% and sensitivities of > 70%. For cerebrovascular disease, these definitions had PPVs of > 80% and sensitivities of> 70%. For other causes of death, PPVs and sensitivities were < 50% for most definitions. Based on these results, we recommend definitions with a combination of “main disease” in both medical and DPC data for cancer and cerebrovascular disease. However, a clear argument cannot be made for other causes of death because of the small sample size. Therefore, the results of this study can be used with confidence for cancer and cerebrovascular disease but should be used with caution for other causes of death.

## Introduction

In recent years, the evaluation of end-of-life care has been widely conducted using claims data [1–5]. Claims data are routinely collected for billing purposes and offer a large longitudinal dataset to researchers [6]. However, for other uses, verifying the validity of the information recorded in the claims data is essential. Claims data are recorded for the purpose of service reimbursement rather than for study purposes and contain little information on the background of patients, examination results, disease severity, and diagnoses [6, 7]. Therefore, researchers must assess the validity and accuracy of key variables such as diagnosis [8]. In the US, Europe, and Asia-Pacific region, claims data have been validated to match medical records or registry data in order to provide some confidence in the use of these data for study purposes [9, 10].

Claims data from the US and Europe are linked at an individual level with data on the use of home care and nursing homes, hospital data, and causes of death [1, 2, 11–13]. In contrast, because the National Database (NDB), which stores claims data for all Japanese citizens, is restricted from being linked to external databases, we cannot ascertain the cause of death from the NDB. Several studies have evaluated the validity of Japanese claims data, including information related to diagnosis [14–17], procedure [14, 18], prescription [19], and discharge [20, 21].

However, the validity of identifying the cause of death in Japanese medical claims data remains unknown. A previous study reviewed the validation of claims data in the Asia-Pacific region [10] and reported that two of forty-three studies evaluated death information. Mealing et al. [22] reported a high sensitivity (92%-99%) and specificity (90.3%-97.9%) of death information by cancer type against the actual death information of a Department of Veterans’ database for patients with cancer in Australia. Sakai et al. [21] reported a moderate to high sensitivity (47%-94%), high specificity (98%-99%), and high PPV (≥95%) for death in claims-based definitions of death. These values correlate with the reason for loss of insured status “died” in enrollment files by inpatients and outpatients among non-dependent persons aged 65–74 years in Japanese workplace health insurance. Additionally, Fujiwara et al. [16] reported a moderate to high sensitivity (75%-100%) and high specificity and PPV (100%) for death in claims-based definitions of death. This result was against the chart review based on electrical medical records of inpatients with cancer in a single hospital in Japan. Although several studies [16, 20, 21] have evaluated death information in the claims data against chart review and enrollment files, there is limited information regarding the method used to identify the cause of death and sensitivity, specificity, and PPV of the cause of death between the death certificate and claims data. The accuracy of the method for identifying the cause of death is important for identifying patients with diseases of interest in end-of-life care. Therefore, this study aimed to verify the validity of the method used to identify the cause of death based on Japanese claims data.

## Materials and methods

### Study design

We conducted this two-site cross-sectional study to validate definitions of the cause of death using information recorded in claims data against the death certificate (the latter serving as the gold standard). This study was reported according to the Standards for Reporting Diagnostic Accuracy (STARD) 2015 statement (S1 Table) [23] and approved by the Institutional Review Board of Tohoku University (Approval No. 2020-1-683; approval date: April 24, 2020).

### Study patients

We included consecutive inpatients aged ≥20 years who died at Tohoku University Hospital or Nagoya University Hospital between January 1, 2018 and December 31, 2019. We obtained death certificates and claims data from each patient’s electronic medical record that was stored by the Center of medical information technology in each university hospital. We linked the death certificate and claims data by a common ID, which is contained in these datasets, using the MERGE statement of DATA step statements (S1 Fig). Exclusion criteria were as follows: 1) absence of a cause of death on the death certificate, 2) death from a disease that could not be the cause of death, 3) death from a cause other than natural causes, and 4) claims data not obtained from the Center of medical information technology in each university hospital.

### Definitions of cause of death based on claims data

Japanese claims data are issued once a month, and they record treatment, prescriptions given for diseases, and discharge information [24, 25]. Japanese claims data are classified into medical and Diagnosis Procedure Combination database (DPC) data [17, 26]. Medical data are claims data issued by most medical institutions and are based on a system whereby points are added after each treatment. DPC data are claims data issued by acute care hospitals and include the cost of each treatment within the daily hospitalization costs set by the Ministry of Health, Labour, and Welfare.

We used two types of disease information from the medical data, in which diseases are classified into two categories: “disease” or “main disease.” “Disease” refers to all diseases recorded. “Main disease” refers to the main flagged disease. In contrast, in the DPC data, disease information is classified into “greatest resource-consuming disease,” “main disease,” “trigger-for-hospitalization disease,” and four other categories [14, 17, 25]. We used “greatest resource-consuming disease,” “main disease,” and “trigger-for-hospitalization disease.” Other categories were not used because they were for comorbidities. Discharge information was recorded as “death,” “cure,” “termination,” or “other.”

We validated nine causes of death (cancer, heart disease, cerebrovascular disease, pneumonia, chronic obstructive pulmonary disease, renal disease, dementia, old age, and infection). The 10th revision of the International Classification of Diseases (ICD-10) codes corresponding to each cause of death is listed in S2 Table. In this study, claims data issued in the month in which the discharge information was “death” were analyzed. This method has been used in previous studies on the NDB [27, 28].

Claims data issued more than 2 months after the patient’s death were excluded because these claims probably indicated that the patient could be alive and that the claims data were issued due to errors such as miscoding discharge/disease status when the claims were issued by the medical institutions [20]. We combined disease categories in the medical or DPC data and the month in which the claims data were issued and created five definitions, which were validated for each cause of death (Table 1). For example, in one case, cause of death was cancer because the discharge information in the medical data in January 2018 was “death” and the “Main disease” was cancer. In a second case, cause of death was cancer and cerebrovascular disease because the discharge information in DPC data in July 2019 was “death,” the “Main disease” was cancer, and the “Greatest resource-consuming disease” was cerebrovascular disease.

**Table 1:** Definitions of causes of death.

Definition	Pattern
1	“Disease” in medical data
	+ claims data issued in the month in which discharge information was “death”
2	“Main disease” in medical data
	+ claims data issued in the month in which discharge information was “death”
3	“Greatest resource-consuming disease” in DPC data
	+ claims data issued in the month in which discharge information was “death”
4	“Main disease” in DPC data
	+ claims data issued in the month in which discharge information was “death”
5	“Trigger-for-hospitalization disease” in DPC data
	+ claims data issued in the month in which discharge information was “death”

Abbreviations; DPC, Diagnosis Procedure Combination.

### Gold standard

In this study, we used the death certificate as the gold standard for the cause of death. In Japanese cause-of-death statistics, the cause of death is identified based on the information in the death certificate.

Two researchers (ST and MM) independently identified the cause of death from information in the death certificates according to guidelines published by the Ministry of Health, Labour, and Welfare in Japan [29, 30]. If the two researchers agreed on the cause of death, it was considered the cause of death. Disagreements were resolved by consensus through discussion.

### Defining true positives, false negatives, false positives, and true negatives

We defined four indices based on previous studies [15, 21]. True positives were defined as cases with any claims-based definition of cause of death (i.e., cause of death obtained from claims) and gold standard definitions of cause of death (i.e., cause of death identified from the death certificate). False negatives were defined as cases with no claims-based definition of cause of death but with a gold standard definition of cause of death. False positives were defined as cases with any claims-based definition of cause of death but no gold standard definitions of cause of death. True negatives were defined as cases with no claims-based definition of cause of death and no gold standard definition of cause of death.

### Statistical analysis

Data related to patient characteristics are presented using standard descriptive statistics of median (interquartile range [IQR]) for continuous variables and number (%) for categorical variables by patients with or without discharge “death” on claims data. We calculated the sensitivity, specificity, PPV, and 95% confidence interval (CI) of each definition. We obtained 95% CIs of these diagnostic indices using the senspec option of PROC FREQ. We listed true positives, false positives, true negatives, and false negatives. Previous studies have emphasized PPVs and sensitivities; we discuss these two measures in this study [31, 32]. S3 Table shows the numbers of true positives, false positives, false negatives, and true negatives for each cause of death. All analyses were performed using SAS software version 9.4 (SAS Institute, Cary, NC).

## Results

### Patients’ characteristics

**Fig 1:**
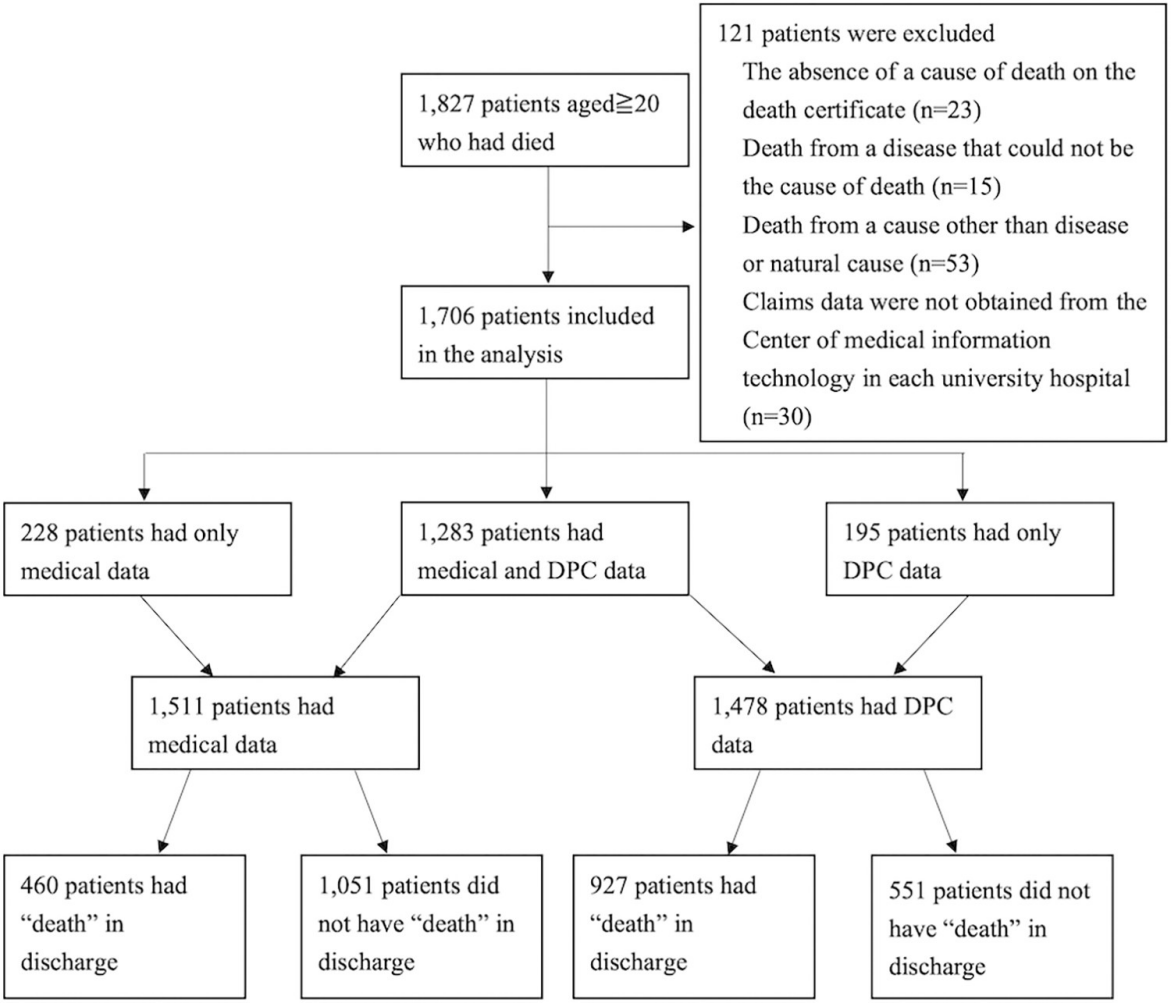
Eligibility flow. Abbreviations; DPC, Diagnosis Procedure Combination.

The eligibility flow is illustrated in Fig 1. After the three exclusion criteria were applied, the final number of patients was 1706 (93.4%). The median (IQR) age of the patients was 71.0 (61.0–79.0) years. The number of patients with both medical and DPC data was 1283 (75.2%). Only 228 patients (13.3%) had medical data only and 195 (11.4%) had DPC data only. Altogether, 1511 patients had medical data and 1478 patients had DPC data. A total of 81.3% (1387/1706 patients) were discharged with “death,” with 30.4% (460/1511 patients) in the medical data and 62.7% (927/1411 patients) in the DPC data. Regarding the characteristics of patients with/without the death information in claims data, patients without the information were more likely to have cancer and die in palliative care units than patients with the information (Table 2).

**Table 2:** Characteristics of patients who fulfilled the eligibility criteria.

		Overall		Patient with discharge ’death’		Patient without discharge “death”	
		n = 1706		n = 1387		n = 319	
Variable	Level	n	%	n	%	n	%
Sex, n(%)	Female	697	40.9	550	39.7	147	46.1
	Male	1009	59.1	828	59.7	181	56.7
Age (median [IQR]), years		71.0 [61.0, 79.0]		71.0 [61.0, 79.0]		70.0 [59.0, 78.0]	
	20–64	543	31.8	428	30.9	115	36.1
	65–74	531	31.1	431	31.1	100	31.3
	75–84	433	25.4	351	25.3	82	25.7
	>85	199	11.7	168	12.1	31	9.7
Length of Stay (median [IQR])		18.0 [7.0, 36.0]		16.0 [6.0, 33.0]		27.00 [10.0, 42.3]	
Hospital, n(%)	Hospital A	1096	64.2	824	59.4	272	85.3
	Hospital B	610	35.8	554	39.9	56	17.6
Place of Death, n(%)	General ward	988	57.9	889	64.1	99	31.0
	Intensive care unit	283	16.6	254	18.3	29	9.1
	Palliative care unit	435	25.5	235	16.9	200	62.7
Cause of Death, n(%)	Cancer	1132	66.4	852	61.4	280	87.8
	Heart disease	117	6.9	107	7.7	10	3.1
	Cerebrovascular disease	74	4.3	67	4.8	7	2.2
	Pneumonia	44	2.6	42	3.0	2	0.6
	COPD	10	0.6	9	0.6	1	0.3
	Renal disease	9	0.5	8	0.6	1	0.3
	Old age	4	0.2	3	0.2	1	0.3
	Dementia	1	0.1	1	0.1	0	0.0
	Infection	37	2.2	34	2.5	3	0.9
	Other	278	16.3	255	18.4	23	7.2

Abbreviations; COPD, Chronic obstructive pulmonary disease; IQR, interquartile range.

The Kappa coefficient was 0.82 when the two researchers identified the cause of death from the death certificates. Based on the gold standard cause of death from the death certificate, the most common cause of death was cancer (66.4%), followed by heart disease (6.9%), cerebrovascular disease (4.3%), and pneumonia (2.6%) (Table 2).

### Accuracy of definitions

For cancer, all definitions had PPVs of > 80%, and Definitions 2–5 had PPVs of > 90% (Table 3). Definitions 1–4 had sensitivities of > 80%, and Definitions 1, 2, and 4 had sensitivities of > 90%. Definition 2–5 had specificities of > 90%. For heart disease, Definitions 2–5 had PPVs of > 50%. Definitions 1, 3, and 4 had sensitivities of > 80%. Definition 2–5 had specificities of > 90%. In cerebrovascular disease, Definitions 2–4 had PPVs of > 70%, and Definitions 2 and 4 had PPVs of > 80%. Definitions 1–4 had sensitivities of > 70%. Definitions 1, 3, and 4 had sensitivities of > 90%. All definitions had specificities of > 90%. For other causes of death, specificities were high, but PPVs and sensitivities were < 50% for most definitions.

**Table 3:** Validity indices for the definitions of the cause of death based on claims data.

Definitions	n	PPV		95% CI				Sensitivity		95% CI				Specificity		95% CI			
Cancer																			
1: medical data	460	0.89	(	0.86	-	0.92	)	1	(	1	-	1	)	0.72	(	0.64	-	0.79	)
2: medical data	460	0.96	(	0.94	-	0.99	)	0.93	(	0.9	-	0.96	)	0.92	(	0.88	-	0.97	)
3: DPC data	927	0.95	(	0.93	-	0.97	)	0.88	(	0.85	-	0.91	)	0.94	(	0.91	-	0.96	)
4: DPC data	927	0.94	(	0.92	-	0.96	)	0.93	(	0.91	-	0.95	)	0.92	(	0.89	-	0.95	)
5: DPC data	927	0.94	(	0.92	-	0.97	)	0.75	(	0.71	-	0.78	)	0.94	(	0.91	-	0.96	)
Heart disease																			
1: medical data	460	0.19	(	0.13	-	0.24	)	1	(	1	-	1	)	0.65	(	0.61	-	0.7	)
2: medical data	460	0.58	(	0.43	-	0.72	)	0.76	(	0.62	-	0.91	)	0.96	(	0.94	-	0.98	)
3: DPC data	927	0.68	(	0.59	-	0.78	)	0.82	(	0.73	-	0.9	)	0.97	(	0.95	-	0.98	)
4: DPC data	927	0.68	(	0.58	-	0.77	)	0.83	(	0.74	-	0.91	)	0.96	(	0.95	-	0.98	)
5: DPC data	927	0.61	(	0.51	-	0.7	)	0.79	(	0.7	-	0.88	)	0.95	(	0.94	-	0.97	)
Cerebrovascular disease																			
1: medical data	460	0.38	(	0.26	-	0.49	)	0.96	(	0.89	-	1	)	0.9	(	0.87	-	0.93	)
2: medical data	460	0.9	(	0.78	-	1	)	0.7	(	0.53	-	0.88	)	1	(	0.99	-	1	)
3: DPC data	927	0.73	(	0.61	-	0.85	)	0.9	(	0.82	-	0.99	)	0.98	(	0.98	-	0.99	)
4: DPC data	927	0.81	(	0.7	-	0.92	)	0.9	(	0.82	-	0.99	)	0.99	(	0.98	-	1	)
5: DPC data	927	0.71	(	0.57	-	0.85	)	0.64	(	0.5	-	0.79	)	0.99	(	0.98	-	0.99	)
Pneumonia																			
1: medical data	460	0.12	(	0.04	-	0.2	)	0.88	(	0.65	-	1	)	0.89	(	0.86	-	0.91	)
2: medical data	460	0.44	(	0.12	-	0.77	)	0.5	(	0.15	-	0.85	)	0.99	(	0.98	-	1	)
3: DPC data	927	0.38	(	0.22	-	0.55	)	0.38	(	0.22	-	0.55	)	0.98	(	0.97	-	0.99	)
4: DPC data	927	0.52	(	0.32	-	0.72	)	0.38	(	0.22	-	0.55	)	0.99	(	0.98	-	0.99	)
5: DPC data	927	0.41	(	0.26	-	0.56	)	0.47	(	0.3	-	0.64	)	0.97	(	0.96	-	0.98	)
Chronic obstructive pulmonary disease (COPD)																			
1: medical data	460	0.02	(	0	-	0.06	)	0.5	(	0	-	1	)	0.9	(	0.87	-	0.93	)
2: medical data	460	-						-						-					
3: DPC data	927	0.4	(	0	-	0.83	)	0.29	(	0	-	0.62	)	1	(	0.99	-	1	)
4: DPC data	927	0.57	(	0.2	-	0.94	)	0.57	(	0.2	-	0.94	)	1	(	0.99	-	1	)
5: DPC data	927	0.2	(	0	-	0.55	)	0.14	(	0	-	0.4	)	1	(	0.99	-	1	)
Renal disease																			
1: medical data	460	0.02	(	0	-	0.05	)	1	(	1	-	1	)	0.87	(	0.84	-	0.9	)
2: medical data	460	0	(	0	-	0	)	0	(	0	-	0	)	0.99	(	0.99	-	1	)
3: DPC data	927	0.2	(	0	-	0.4	)	0.43	(	0.06	-	0.8	)	0.99	(	0.98	-	0.99	)
4: DPC data	927	0.24	(	0.03	-	0.44	)	0.57	(	0.2	-	0.94	)	0.99	(	0.98	-	0.99	)
5: DPC data	927	0.13	(	0	-	0.29	)	0.29	(	0	-	0.62	)	0.98	(	0.98	-	0.99	)
Dementia																			
1: medical data	460	-						-						-					
2: medical data	460	-						-						-					
3: DPC data	927	-						-						-					
4: DPC data	927	-						-						-					
5: DPC data	927	0	(	0	-	0	)	0	(	0	-	0	)	1	(	1	-	1	)
Old age																			
1: medical data	460	-						-						-					
2: medical data	460	-						-						-					
3: DPC data	927	-						-						-					
4: DPC data	927	-						-						-					
5: DPC data	927	-						-						-					
Infection																			
1: medical data	460	0.04	(	0.01	-	0.06	)	0.88	(	0.65	-	1	)	0.6	(	0.56	-	0.65	)
2: medical data	460	0.2	(	0	-	0.4	)	0.38	(	0.04	-	0.71	)	0.97	(	0.96	-	0.99	)
3: DPC data	927	0.29	(	0.15	-	0.43	)	0.44	(	0.26	-	0.63	)	0.97	(	0.96	-	0.98	)
4: DPC data	927	0.38	(	0.2	-	0.56	)	0.41	(	0.22	-	0.59	)	0.98	(	0.97	-	0.99	)
5: DPC data	927	0.2	(	0.04	-	0.36	)	0.19	(	0.04	-	0.33	)	0.98	(	0.97	-	0.99	)

CI of diagnostic indices was obtained using the senspec option of PROC FREQ.

Abbreviations; DPC, Diagnosis Procedure Combination; PPV, positive predictive value; 95% CI, 95% confidence interval.

## Discussion

To the best of our knowledge, this multicenter cross-sectional study is the first to develop and validate definitions to identify the cause of death in Japanese claims data. Although several studies have evaluated the validity of information such as diagnosis [14–17], procedure [14, 18], prescription [19], and discharge information [20, 21] in Japanese claims data, there has been limited information regarding the method used to identify the cause of death. The major finding of this study was that the cause of death due to cancer and cerebrovascular disease in Japanese claims could be satisfactorily identified using definitions where PPVs were 89%-96% in cancer and 71%-90% in cerebrovascular disease. Contrary to a previous study that evaluated sudden cardiac death [33], our results for heart diseases showed low PPV. This was because the participants were patients with opioid prescriptions who were validated based on chart review and death certificates in the previous study. Additionally, our results showed that the sensitivity and PPV for the “Main disease” in the DPC were more likely to be accurate than those for the “Greatest resource-consuming disease” in the DPC. While the cause of death from the death certificate was likely to match with the “Main disease” in the DPC, which was a condition given by the physicians, it might show a different trend from the “Greatest resource-consuming disease” in the DPC, which was a condition responsible for the greatest use of medical resources for several characteristics, such as diagnoses, comorbidities, and complications, given by the physicians [17]. Therefore, based on our results, we make two recommendations for future studies examining end-of-life care using the Japanese claims database. First, using medical data, it might be useful to use Definition 2 (“Main disease” in medical data + claims data issued in the month in which discharge information was “death”): for cancer, heart disease, and cerebrovascular disease, the ranges of PPVs and sensitivities were 56.8–96.4% and 70.4–93.1%, respectively. Second, using DPC data, it might be useful to use Definition 4 (“Main disease” in DPC data + claims data issued in the month in which discharge information was “death’): for cancer, heart disease, and cerebrovascular disease, the ranges of PPVs and sensitivities were 67.0–94.2% and 82.4–93.2%, respectively.

The interesting finding of this study was that approximately 20% of patients were missing on from the discharge with “death” data and reported characteristics. Compared to previous studies [20, 21], we showed a different proportion of missing death information among inpatients. Sakai et al. [21] reported death information was sensitivity of 94.4% by inpatients and 47.4% by outpatients. Medical institutions may omit recording of patient deaths in the claims data due to lack of motivation, as these data are primarily collected for reimbursement purposes. Patients without death information in the claims data were more likely to have cancer and die in palliative care units, than patients with information in the claims data. Additionally, there were no palliative care units at hospital B. Thus, it might vary among departments and hospitals. A previous study showed similar findings regarding the difference in results of the diagnosis of comorbidities between four hospitals [14]. However, our study corroborates the proportion of missing death information and the characteristics of patients with those shown previously, which we believe will be useful for future database studies.

The findings of this study can be applied to the NDB. Several studies have assessed end-of-life care using the NDB in Japan [27, 28, 34, 35]. Even though the accuracy of the method used in previous studies to identify the cause of death is important for the identification of patients with diseases of interest in end-of-life care, the method has not been validated. Therefore, the results of this study can contribute to a more accurate identification of terminal patients in future studies on end-of-life care using the NDB.

### Limitations

This study had several limitations. First, the sample size may have been insufficient. Although we used death certificates for 2 years and claims data from two institutions, more than half of the deaths were from cancer, and the sample size for other diseases was small. Therefore, it is necessary to increase the sample size for each cause of death in future studies.

Second, a clear definition cannot be recommended for patients with both medical and DPC data. The results of this study can be applied to patients with medical or DPC data. However, PPVs for Definitions 2 and 4 do not differ significantly; therefore, it is possible to identify patients using either definition, although a new definition needs to be developed.

Third, there might be measurement errors in the information on death certificates as the gold standard. For example, Mieno et al. [29] evaluated that the concordance rate was relatively high for cancer (81%) but low for heart disease (55%) and pneumonia (9%), which reported on death certificates against a reference standard of pathologist assessment based on autopsy data and clinical records. Therefore, the cause of death identified in this study might not be the true cause due to measurement error. However, it was difficult to obtain pathological autopsy results as the gold standard for the sample size of this study. Therefore, in this study, we considered death certificates as a plausible gold standard.

Finally, the variables used in this study were insufficient. In this study, only the disease categories and discharge information were used. Japanese claims data include other information such as treatment details and hospital charges. Sato et al. [15] identified breast cancer with a high probability in Japanese claims data by combining breast cancer diagnosis and treatment procedure codes. In the future, it will be necessary to search for variables that improve the accuracy of each cause of death and to conduct analyses that include these variables.

## Conclusions

This study validated a method for identifying the cause of death using Japanese claims data with sufficient accuracy. The results showed that for cancer, heart disease, and cerebrovascular disease, Definition 2 in the medical data and Definition 4 in the DPC data exhibited high PPV and sensitivity. For heart disease, PPV was below 70% for all definitions. Also, we could not make a clear argument for other causes of death because of the lack of samples. Therefore, the definitions of cause of death using the claims data identified in this study can be used with confidence for cancer and cerebrovascular disease but should be used with caution for other causes of death.

## Supporting information

S1 Fig Data flow. (TIF)

S1 Table STARD 2015 checklist. (DOCX)

S2 Table The 10th revision of the International Classification of Diseases (ICD-10) codes corresponding to each cause of death. (DOCX)

S3 Table Number of true positives, false positives, false negatives, and true negatives for each cause of death. (DOCX)

S1 File Software source code. (ZIP)
