# Supplementary figures and images for: Validation study on definition of cause of death in Japanese claims data
Source: PLoS One. 2023 Mar 23;18(3):e0283209. doi: 10.1371/journal.pone.0283209 (PMC10035912; doi:10.1371/journal.pone.0283209)

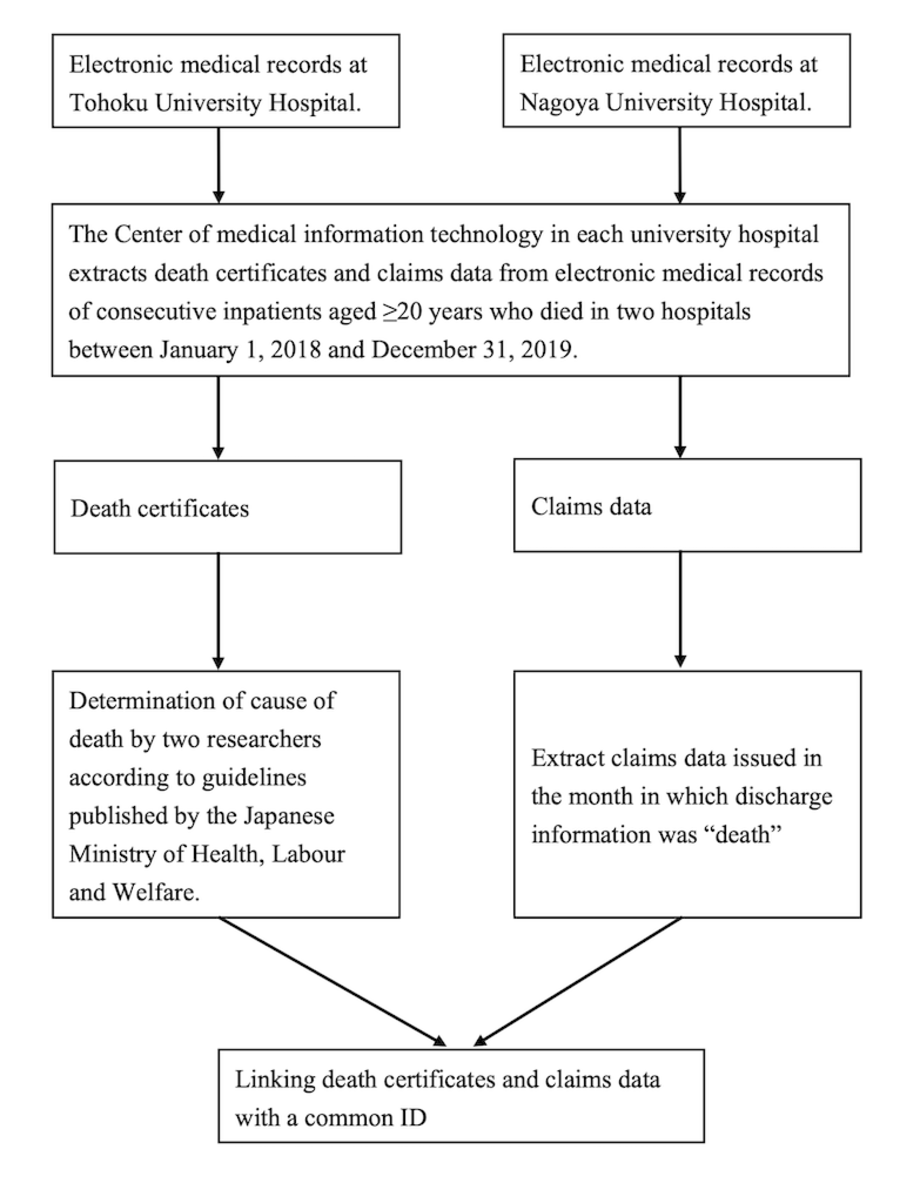

Supplement: S1 Fig — (TIF) [file pone.0283209.s001.tif]
