# Supplementary material for: Validation study on definition of cause of death in Japanese claims data
Source: PLoS One. 2023 Mar 23;18(3):e0283209. doi: 10.1371/journal.pone.0283209 (PMC10035912; doi:10.1371/journal.pone.0283209)
Supplement: S2 Table — (DOCX) [file pone.0283209.s003.docx]

S2 Table. The 10th revision of the International Classification of Diseases (ICD-10) codes corresponding to each cause of death

| Cause of death |  | ICD-10 codes |
| --- | --- | --- |
| Cancer |  | C00-C97 |
| Heart disease |  | I01-I02.0, I05-I09, I20-I25, I27, I30-I52 |
| Cerebrovascular disease |  | I60-I69 |
| Pneumonia |  | J12-J18 |
| Chronic obstructive pulmonary disease (COPD) |  | J41-J44 |
| Renal disease |  | N17-N19 |
| Dementia |  | F01-F03, G30 |
| Old age |  | R54 |
| Infection |  | A00-B99 |
