# Supplementary material for: Validation study on definition of cause of death in Japanese claims data
Source: PLoS One. 2023 Mar 23;18(3):e0283209. doi: 10.1371/journal.pone.0283209 (PMC10035912; doi:10.1371/journal.pone.0283209)
Supplement: S3 Table — (DOCX) [file pone.0283209.s004.docx]

S3 Table. Number of true positives, false positives, false negatives, and true negatives for each cause of death

| Definitions |  | n |  | True positive |  | False positive |  | False negative |  | True negative |
| --- | --- | --- | --- | --- | --- | --- | --- | --- | --- | --- |
|  |  |  |  |  |  |  |  |  |  |  |
| Cancer | | | | | | | | | | |
| 1: medical data |  | 460 |  | 319 |  | 40 |  | 0 |  | 101 |
| 2: medical data |  | 460 |  | 296 |  | 11 |  | 23 |  | 130 |
| 3: DPC data |  | 927 |  | 472 |  | 24 |  | 64 |  | 367 |
| 4: DPC data |  | 927 |  | 498 |  | 31 |  | 38 |  | 360 |
| 5: DPC data |  | 927 |  | 400 |  | 24 |  | 136 |  | 367 |
| Heart disease | | | | | | | | | | |
| 1: medical data |  | 460 |  | 34 |  | 147 |  | 0 |  | 279 |
| 2: medical data |  | 460 |  | 26 |  | 19 |  | 8 |  | 407 |
| 3: DPC data |  | 927 |  | 62 |  | 29 |  | 14 |  | 822 |
| 4: DPC data |  | 927 |  | 63 |  | 30 |  | 13 |  | 821 |
| 5: DPC data |  | 927 |  | 60 |  | 39 |  | 16 |  | 812 |
| Cerebrovascular disease | | | | | | | | | | |
| 1: medical data |  | 460 |  | 26 |  | 43 |  | 1 |  | 390 |
| 2: medical data |  | 460 |  | 19 |  | 2 |  | 8 |  | 431 |
| 3: DPC data |  | 927 |  | 38 |  | 9 |  | 4 |  | 871 |
| 4: DPC data |  | 927 |  | 38 |  | 9 |  | 4 |  | 876 |
| 5: DPC data |  | 927 |  | 27 |  | 11 |  | 15 |  | 874 |
| Pneumonia | | | | | | | | | | |
| 1: medical data |  | 460 |  | 7 |  | 52 |  | 1 |  | 400 |
| 2: medical data |  | 460 |  | 4 |  | 5 |  | 4 |  | 447 |
| 3: DPC data |  | 927 |  | 13 |  | 21 |  | 21 |  | 872 |
| 4: DPC data |  | 927 |  | 13 |  | 12 |  | 21 |  | 881 |
| 5: DPC data |  | 927 |  | 16 |  | 23 |  | 18 |  | 870 |
| Chronic obstructive pulmonary disease (COPD) | | | | | | | | | | |
| 1: medical data |  | 460 |  | 1 |  | 47 |  | 1 |  | 411 |
| 2: medical data |  | 460 |  | 0 |  | 0 |  | 2 |  | 458 |
| 3: DPC data |  | 927 |  | 2 |  | 3 |  | 5 |  | 917 |
| 4: DPC data |  | 927 |  | 4 |  | 3 |  | 3 |  | 917 |
| 5: DPC data |  | 927 |  | 1 |  | 4 |  | 6 |  | 916 |
| Renal disease | | | | | | | | | | |
| 1: medical data |  | 460 |  | 1 |  | 60 |  | 0 |  | 399 |
| 2: medical data |  | 460 |  | 0 |  | 3 |  | 1 |  | 456 |
| 3: DPC data |  | 927 |  | 3 |  | 12 |  | 4 |  | 908 |
| 4: DPC data |  | 927 |  | 4 |  | 13 |  | 3 |  | 907 |
| 5: DPC data |  | 927 |  | 2 |  | 14 |  | 5 |  | 906 |
| Dementia | | | | | | | | | | |
| 1: medical data |  | 460 |  | 0 |  | 7 |  | 0 |  | 453 |
| 2: medical data |  | 460 |  | 0 |  | 0 |  | 0 |  | 460 |
| 3: DPC data |  | 927 |  | 0 |  | 0 |  | 1 |  | 926 |
| 4: DPC data |  | 927 |  | 0 |  | 0 |  | 1 |  | 926 |
| 5: DPC data |  | 927 |  | 0 |  | 1 |  | 1 |  | 925 |
| Old age | | | | | | | | | | |
| 1: medical data |  | 460 |  | 0 |  | 1 |  | 0 |  | 459 |
| 2: medical data |  | 460 |  | 0 |  | 1 |  | 0 |  | 459 |
| 3: DPC data |  | 927 |  | 0 |  | 0 |  | 3 |  | 924 |
| 4: DPC data |  | 927 |  | 0 |  | 0 |  | 3 |  | 924 |
| 5: DPC data |  | 927 |  | 0 |  | 0 |  | 3 |  | 924 |
| Infection | | | | | | | | | | |
| 1: medical data |  | 460 |  | 7 |  | 180 |  | 1 |  | 272 |
| 2: medical data |  | 460 |  | 3 |  | 12 |  | 5 |  | 440 |
| 3: DPC data |  | 927 |  | 12 |  | 29 |  | 15 |  | 871 |
| 4: DPC data |  | 927 |  | 11 |  | 18 |  | 16 |  | 882 |
| 5: DPC data |  | 927 |  | 5 |  | 20 |  | 22 |  | 880 |

Abbreviations; DPC, Diagnosis Procedure Combination.
